# Chongcao-Shencha Attenuates Liver and Kidney Injury through Attenuating Oxidative Stress and Inflammatory Response in D-Galactose-Treated Mice

**DOI:** 10.1155/2016/3878740

**Published:** 2016-06-02

**Authors:** Cailan Li, Zhizhun Mo, Jianhui Xie, Lieqiang Xu, Lihua Tan, Dandan Luo, Hanbin Chen, Hongmei Yang, Yucui Li, Ziren Su, Zuqing Su

**Affiliations:** ^1^School of Chinese Materia Medica, Guangzhou University of Chinese Medicine, Guangzhou 510006, China; ^2^Guangdong Provincial Key Laboratory of Clinical Research on Traditional Chinese Medicine Syndrome, The Second Affiliated Hospital, Guangzhou University of Chinese Medicine, Guangzhou 510120, China; ^3^The Second Affiliated Hospital, Guangzhou University of Chinese Medicine, Guangzhou 510120, China

## Abstract

The Chongcao-Shencha (CCSC), a Chinese herbal compound formula, has been widely used as food material and medicine for enhancing physical strength. The present study investigated the possible effect of CCSC in alleviating the liver and kidney injury in D-galactose- (D-gal-) treated mice and the underlying mechanism. Mice were given a subcutaneous injection of D-gal (200 mg/kg) and orally administered CCSC (200, 400, and 800 mg/kg) daily for 8 weeks. Results indicated that CCSC increased the depressed body weight and organ index induced by D-gal, ameliorated the histological deterioration, and decreased the levels of ALT, AST, BUN, and CRE as compared with D-gal group. Furthermore, CCSC not only elevated the activities of antioxidant enzymes SOD, CAT, and GPx but also upregulated the mRNA expression of SOD1, CAT, and GPx1, while decreasing the MDA level in D-gal-treated mice. Results of western blotting analysis showed that CCSC significantly inhibited the upregulation of expression of nuclear factor kappa B (NF-*κ*B) p65, p-p65, p-I*κ*B*α*, COX2, and iNOS and inhibited the downregulation of I*κ*B*α* protein expression caused by D-gal. This study demonstrated that CCSC could attenuate the liver and kidney injury in D-gal-treated mice, and the mechanism might be associated with attenuating oxidative stress and inflammatory response.

## 1. Introduction

Researches have demonstrated that chronic administration of D-gal induced changes resembling natural aging in rodents [1–3]. In animals, D-gal is normally metabolized by D-galactokinase and galactose-1-phosphate uridyltransferase, but oversupply of D-gal results in its abnormal metabolism and induces oxidative stress, inflammation response, and tissue damage, including the brain, liver, and kidney [4, 5]. It is observed that there is high production of reactive oxygen species (ROS) and low activities of antioxidant enzymes in the body fluids and tissues in D-gal-treated rats [6–8]. Since then, D-gal injection has been gradually used to establish an aging model for antiaging or organ injury research.

Oxidative damage caused by ROS is expected to play a role in D-gal-induced age-related changes in tissues [9]. The excess ROS formation can damage cellular macromolecules such as lipids, proteins, and DNA and cause cell damage [10, 11]. However, the endogenous antioxidant system, including superoxide dismutases (SOD), catalase (CAT), and glutathione peroxidase (GPx), is especially necessary to scavenge the excess ROS and meanwhile may cause the changes of some biomarkers [12]. In addition, it has been well demonstrated that inflammation is one of a variety of biological phenomena caused by oxidative stress [13]. Nuclear factor kappa B (NF-*κ*B) is a ubiquitous transcription factor critically involved in the proinflammatory response. And active NF-*κ*B is also one of the most important regulators of transcription of response genes encoding inflammation associated enzymes such as cyclooxygenase (COX2) and nitric oxide synthase (iNOS), which were extensively studied in inflammation [14].

Herbal formula is characteristic of multiple herbs and has versatile pharmacological activities based on the single effect of multiple herbs as well as the compatibility effect of multiple traditional Chinese medicines, which is considered to play an important role in the treatment of diseases. CCSC, a Chinese herbal compound formula, consists of five traditional Chinese herbs, including Misai Kuching (*Clerodendranthus spicatus* (Thunb.), C.Y.Wu), North* Cordyceps* (*Cordyceps militaris *L. Link), Longan Arillus (*Dimocarpus longan *Lour.), Folium Ginseng (*Panax ginseng* C. A. Mey.), and Corni Fructus (*Cornus officinalis* Sieb. et Zucc.). The five herbs are widely used as food material and medicine in the clinic and daily life for the effect of  “kidney-reinforcing” and “enhancing physical strength.” Recent researches found that the five herbs have a variety of pharmacological activities, including antioxidant, antiaging, hepatoprotective, and immunomodulatory effects [15–18]. It should be noted that traditional herbs and spices also exist along with their active constituents. Researchers found that CCSC contained various active components, such as ginsenoside, loganin, cordycepin, rosmarinic acid, and uridine, which showed a variety of pharmacological activities, including antioxidant [19, 20], anti-inflammatory [21], and antifatigue [22]. However, no endeavor to date has been made to investigate the possible ameliorative effect of CCSC in liver and kidney injury induced by D-gal.

In this study, we investigated the possible effect of CCSC in alleviating the liver and kidney injury in D-gal-treated mice by measuring the body weight, organ index, and histological lesion. Additionally, the effects of CCSC on oxidative stress-related enzyme levels and mRNA expression, the protein expression of iNOS, COX2, and p-p65, p65, p-I*κ*B*α*, and I*κ*B*α* in NF-*κ*B signaling pathway were investigated to illuminate the possible underlying mechanisms.

## 2. Materials and Methods

### 2.1. Drug and Chemical Reagents

Misai Kuching, North Cordyceps, Longan Arillus, Folium Ginseng, and Corni Fructuswere purchased from the Yunnan Chinese Herbal Medicine Department. These herbs were authenticated by one of our authors, Professor Ziren Su, Guangzhou University of Chinese Medicine (GZUCM). The voucher specimens (Yu 15-6-25) were kept at School of Chinese Materia Medica, GZUCM, for reference. D-gal and vitamin E (VE) were purchased from Sigma-Aldrich (St. Louis, USA). Commercial biochemical assay kits for the measurements of superoxide dismutases (SOD), catalase (CAT), glutathione peroxidase (GPx), malondialdehyde (MDA), alanine aminotransferase (ALT), aspartate transaminase (AST), blood urea nitrogen (BUN), and creatinine (CRE) were purchased from Nanjing Jiancheng Bioengineering Institute (Nanjing, China). Tween 80 was purchased from Sinopharm (Shanghai, China). All reagents used were of either analytical or chromatographic grade.

### 2.2. Preparation of Plant Extract

The mixtures (CCSC) of dried Misai Kuching (300 g), North Cordyceps (100 g), Longan Arillus (300 g), Corni Fructus (250 g), and Folium Ginseng (150 g) were ground into powders and soaked in distilled water at room temperature for 30 min, followed by refluxing with distilled water (ratio: 1 g/10 mL) for 2 h. After filtration, the extraction procedure was repeated twice. The filtered extracts were pooled and evaporated under reduced pressure. To analyze the chemical composition, CCSC was dissolved in 50% methanol. For pharmacological tests, CCSC was dissolved in distilled water containing 1% Tween 80 solution.

### 2.3. HPLC-Electrospray Ionization-MS Analysis

HPLC experiments were conducted on a Shimadzu (Kyoto, Japan) HPLC system consisting of an LC-20AD quaternary solvent delivery system, an SIL-20AC autosampler, and a CTO-20AC column oven. The extracts were mixed with methanol and water (ratio: 1 : 1 v/v) and filtered through 0.22 *μ*m microporous membrane prior to high performance liquid chromatography-electrospray ionization-MS (HPLC-ESI-MS/MS) analysis. The Kromasil KR100-5C18 column (250 mm × 4.6 mm, E17096) was applied for chromatographic separations. The mobile phase was composed of A (acetonitrile) and B (water) with a linear gradient elution: 0–20 min, 5–10% A; 20–40 min, 10–18% A; 40–65 min, 18% A; 65–80 min, 18–30% A; 80–85 min, 30–35% A; 85–95 min, 35–40% A; 95–100 min, 40–45% A; 100–110 min, 45–90%; 110–120 min, 90%. The injection volume was 10 *μ*L at a flow rate of 0.4 mL/min. The column oven temperature was maintained at 25°C.

A Triple TOF*™* 5600 system equipped with an electrospray ionization (ESI) source for mass detection and data processing was achieved with Multiquant*™* Software system (AB SCIEX, CA, USA). The following parameter settings were used: ion spray voltage floating, −4500 kV; ion source heater, 500°C; curtain gas, 35 psi; ion source gas 1, 55 psi; and ion source gas 2, 55 psi. Mass analyzer scanned from 100 to 1500* mz*. The MS-MS spectra were recorded in auto-MS-MS mode. The CE was −5 eV, and the CP was −100 eV in the MS/MS experiment. Mass spectra were simultaneously acquired using electrospray ionization in the positive and negative ionization modes.

### 2.4. Animals and Treatment Procedure

Male Kunming (KM) mice (18–22 g) were obtained from the Laboratory Animal Services Centre of GZUCM. After acclimatization for one week under constant conditions of temperature (23 ± 1°C) and humidity (40–60%) on a 12 h light/dark cycle with free access to food and water, the animals were randomly divided into 6 groups (*n* = 10): control group, D-gal group (200 mg/kg), CCSC-L group (200 mg/kg), CCSC-M group (400 mg/kg), CCSC-H group (800 mg/kg), and VE group (80 mg/kg, the positive control). Except for the control, mice received a daily subcutaneous injection of D-gal at a dose of 200 mg/kg, while those in the control group received an injection of the same volume of physiological saline (0.9% NaCl). At the same time, mice in the treatment groups were administered orally with CCSC (200, 400, and 800 mg/kg/day) and VE (80 mg/kg/day) dissolved in distilled water containing 1% Tween 80 solution, respectively. Meanwhile, mice in the control and D-gal groups were given an equal volume of distilled water containing 1% Tween 80 solution without CCSC. All drugs (CCSC, VE, and D-gal) and vehicle were given daily to the animals between 4:30 a.m. and 6:00 a.m. for 8 weeks in a volume of 10 mL/kg body weight. The body weight and food intake of the animals were measured weekly.

### 2.5. Body Weight and Organ Indexes Measurement

During the entire experiment, body weights were measured every week. After 8 weeks, the mice were sacrificed, and the spleens, thymi, kidneys, and livers were carefully dissected out, washed with cold sterile physiological saline, and weighed. Their weights relative to the final body weight were calculated as organ indexes. Then, the liver and kidney were stored immediately at −80°C for the sequent biochemical measurements, western blotting analysis, and quantitative real-time PCR analysis.

### 2.6. Biochemical Assays

For biochemical analysis, 10% (w/v) tissue homogenate was prepared in sodium phosphate buffer (0.1 M PBS, pH 7.4) containing a protease inhibitor cocktail (Sigma-Aldrich), using a homogenizer at the speed of 4000 rpm for 3 min. The homogenate was centrifuged at 3000 rpm for 15 min at 4°C. The supernatants were collected for biochemical analysis. The protein concentrations were measured by BCA (bicinchoninic acid) method using bovine serum albumin as a standard. The activities of SOD, CAT, and GPx, as well as the levels of MDA in liver and kidney, were determined by the assay kit according to its provider's instructions.

### 2.7. Determination of Liver and Kidney Functions

Blood samples were collected from the mice eye socket after 8 weeks. The plasma was prepared by centrifugation at 3000 rpm for 10 min at 4°C. An automatic biochemistry analyzer (RT-2100, Rayto Shenzhen Rayto Life Science Co., Ltd., China) was used to measure the contents of ALT, AST, CRE, and BUN in serum according to the assay kit providers' instructions.

### 2.8. Quantitative Real-Time PCR Analysis

Total RNA was extracted from liver and kidney tissues using Trizol Reagent (Invitrogen Life Technologies, Carlsbad, CA, USA) according to the manufacturer's instructions. The total RNA was digested with RNase-free DNase for 30 min at 42°C. Two *μ*g of the total RNA was used for cDNA synthesis using real-time quantitative PCR SYBR Green Master (Rox). Concentrations of reagents used were determined according to the manufacturer's instructions. The transcript of the constitutive gene 18S was used as an internal control. The sequences of the primers were listed in Table 1. The reaction mixture was subjected to PCR to amplify the sequences to obtain the desired primers. Amplification was performed with 45 cycles of denaturation at 95°C for 10 s, annealing at 60°C for 30 s, and extension at 72°C for 30 s. Relative gene expression was calculated by 2^−ΔΔCT^ method using cycle time values and data for normalization. The changes in the gene expression ratio were calculated using BioPhotometer plus data analysis software.

### 2.9. Western Blotting Analysis

The tissue proteins were separated by electrophoresis on sodium dodecyl sulfate- (SDS-) polyacrylamide gels and transferred to polyvinylidene difluoride (PVDF) membranes (Millipore, Temecula, CA) using a semidry transfer system. The membranes were first incubated in blocking solution (5% skim milk) and then incubated overnight at 4°C with the primary antibodies of iNOS, COX2, I*κ*B*α*, p-I*κ*B*α*, p65, p-p65, and *β*-actin (Sigma, St. Louis, MO, USA). The *β*-actin was used as internal control. Immunoblots were incubated with the corresponding secondary antibody conjugated with horseradish peroxidase for 1 h. Membranes were developed using an electrochemiluminescence kit (Merck, China). The density of the immunoreactive bands was analyzed using Image J software (National Institutes of Health, Bethesda, MD, USA).

### 2.10. Histopathological Assessment

For histological assessment, the liver and kidney tissues were fixed in formalin and then embedded in paraffin. Tissues 5 *μ*m thick were taken, placed onto glass slides, deparaffinized, and stained with hematoxylin eosin (H&E). All tissue sections were observed under a microscope (Nikon Corporation, Tokyo, JP).

### 2.11. Statistical Analysis

Statistical analysis was performed with GraphPad Prism 5 (GraphPad Software, Inc.) software. Data were expressed as means ± standard deviation (SD). Differences among groups were analyzed by one-way analysis of variance (ANOVA) followed by Dunnett's test. A value of *P* < 0.05 or *P* < 0.01 was considered statistically significant.

## 3. Results

### 3.1. Separation and Identification Compounds of CCSC with HPLC-ESI-MS/MS

**Figure 1:**
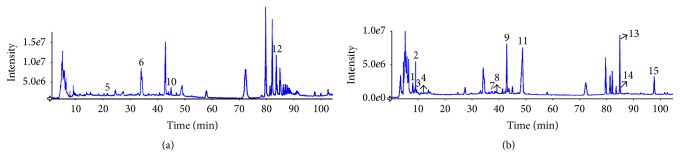
HPLC-ESI-MS/MS base peak chromatograms of the aqueous extracts of the CCSC recorded at positive ion mode (a) and negative ion mode (b). Peak numbers follow those listed in Table 2.

The HPLC-ESI-MS/MS analysis was carried out in both negative and positive ionization modes. The HPLC-ESI base peak chromatograms in the positive mode (a) and negative mode (b) are shown in Figure 1. The identities, retention times (RT), molecular weight and observed molecular ions ([M + H]^+^ *mz*/[M − H]^−^ *mz*), and fragment ions for individual compounds are presented in Table 2. By comparing their retention time and recorded literatures, 15 compounds were identified. Peaks numbered 1–15 represented salvianic acid, gallic acid, uridine, guanosine, cordycepin, verbenalin, caffeic acid, baicalein, loganin, sweroside, rosmarinic acid, 2*α*-hydroxy-ursolic acid, ginsenoside Rg1, ginsenoside Re, and methyl rosmarinate, respectively.

### 3.2. Effect of CCSC on Body Weights and Organ Indexes

As shown in Table 3, at the beginning of the experiment, we did not find any differences in the mean body weight compared with the control mice (*P* > 0.05). However, at the end of the experiment, the mean body weight of D-gal-treated mice was significantly lower than the control, CCSC, and VE treatment groups. Organ indexes, including liver, kidney, thymus, and spleen, showed significant decrease in the D-gal-treated mice compared with control mice (*P* < 0.05 or *P* < 0.01). Administration of CCSC and VE restored the liver, kidney, thymus, and spleen indexes in a dose-dependent manner. These results suggested that CCSC could improve body weight and organ condition of D-gal-treated mice.

### 3.3. Effect of CCSC on Biochemistry Index

**Figure 2:**
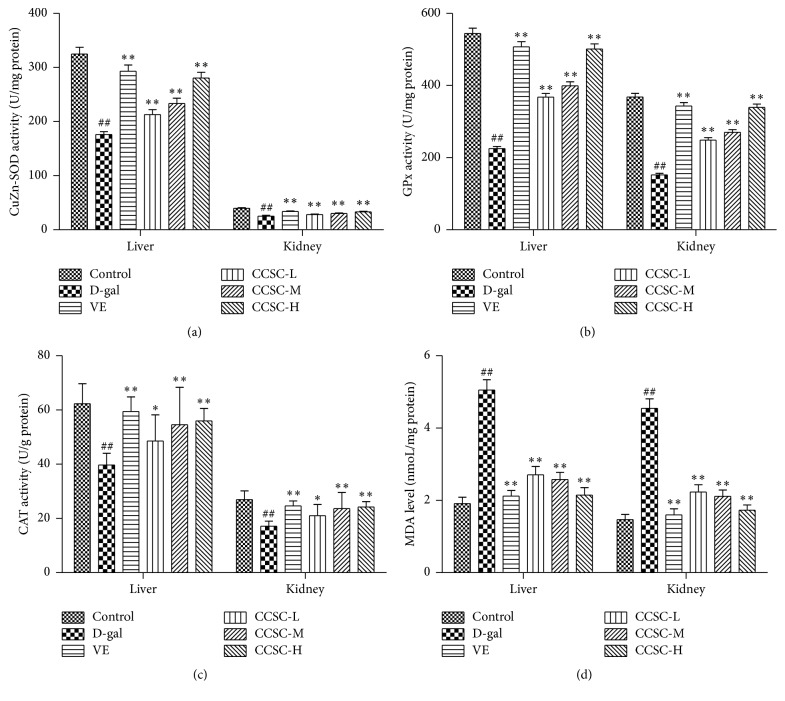
Effect of CCSC on the SOD (a), GPx (b), and CAT (c) activities and MDA (d) content in the liver and kidney of the D-gal-treated mice. Results are expressed as a mean ± SD (*n* = 10). ^##^ *P* < 0.01 as compared with the control group. ^*∗*^ *P* < 0.05 and ^*∗∗*^ *P* < 0.01 as compared with the D-gal group.

As shown in Figures 2(a), 2(b), and 2(c), the SOD, CAT, and GPx activities of liver and kidney in the D-gal group decreased significantly (*P* < 0.05) compared with the control group. Moreover, our results (Figure 2(d)) also showed that the level of MDA in mice liver and kidney in the D-gal treatment group was significantly higher than that in the control group (*P* < 0.01). However, the SOD, CAT, and GPx activities were significantly (*P* < 0.01) restored and the MDA level was attenuated by CCSC and VE in a dose-dependent manner. The results suggested that CCSC treatment could significantly improve SOD, CAT, and GPx activities and decreased MDA level in D-gal-treated mice.

### 3.4. Quantitative Real-Time PCR Analysis

**Figure 3:**
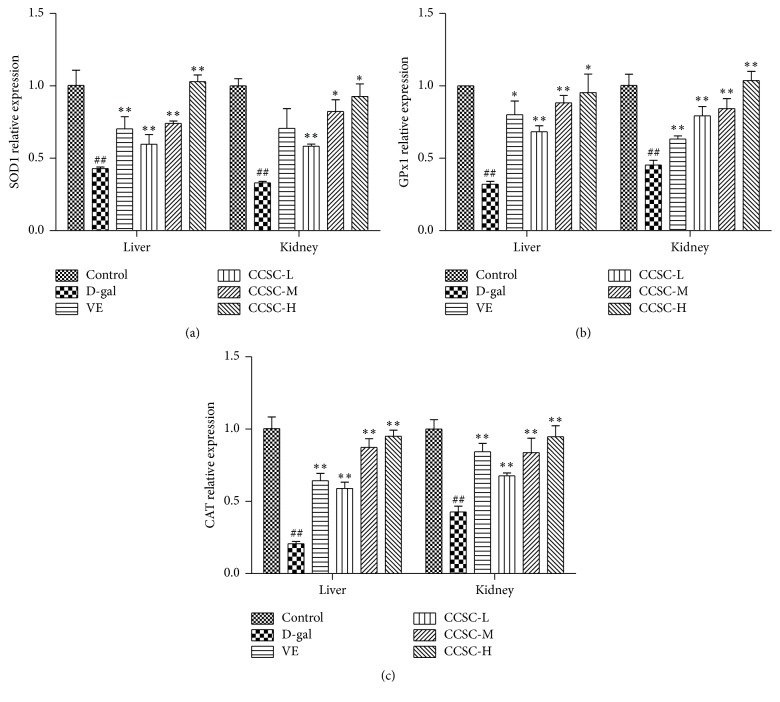
Effect of CCSC on mRNA expressions of SOD1 (a), CAT (b), and Gpx1 (c) in the liver and kidney of the D-gal-treated mice. Results are expressed as a mean ± SD (*n* = 10). ^##^ *P* < 0.01 as compared with the control group. ^*∗*^ *P* < 0.05 and ^*∗∗*^ *P* < 0.01 as compared with the D-gal group.

As for the gene expression, SOD1, CAT, and GPx were significantly downregulated in the D-gal group compared with the control group, while they were significantly upregulated after administration of CCSC in a dose-dependent manner (*P* < 0.05 or *P* < 0.01, Figure 3). As a conventional antioxidant, VE was also observed to significantly upregulate these related gene expressions in D-gal-treated mice. Interestingly, the result showed that both CCSC-M (400 mg/kg) and CCSC-H (800 mg/kg) groups showed more potent effects in upregulating the hepatic and renal mRNA expressions of SOD1, CAT, and GPx than VE group.

### 3.5. Determinations of ALT, AST, BUN, and CRE Levels

Table 4 showed that the levels of AST, ALT, CRE, and BUN significantly increased after D-gal treatment compared with the control group. However, this increase was significantly suppressed by CCSC and VE (*P* < 0.05 or *P* < 0.01) treatment. In addition, CCSC-L (200 mg/kg) exhibited superior suppressive effect on AST and ALT levels in liver than CCSC-M (400 mg/kg) and CCSC-H (800 mg/kg), while, in kidney, CCSC significantly suppressed the levels of CRE and BUN in a dose-dependent manner.

### 3.6. Western Blotting Analysis

**Figure 4:**
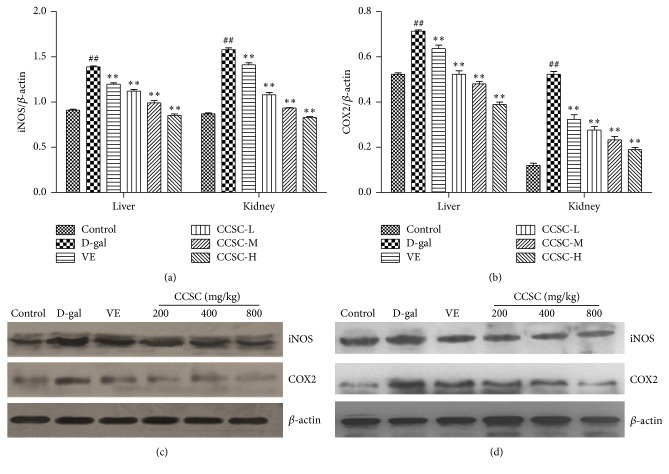
Western blotting analysis of iNOS and COX2 protein expression in the liver and kidney tissues. Relative density analysis of iNOS (a) and COX2 (b) and the representative images of expression of iNOS and COX2 in the liver (c) and kidney (d) tissues. The relative protein level between the tested target protein and internal standard *β*-actin was calculated and labeled on the *y*-axis. The bands are from a representative blot. Lane-1: control group; lane-2: D-gal-treated group; lane-3: VE-treated group (80 mg/kg); lane-4: CCSC-L-treated group (200 mg/kg); lane-5: CCSC-M-treated group (400 mg/kg); lane-6: CCSC-H-treated group (800 mg/kg). Values are expressed as mean ± SD in each group. ^##^ *P* < 0.01 as compared with the control group; ^*∗∗*^ *P* < 0.01 as compared with the D-gal group, *n* = 3.

**Figure 5:**
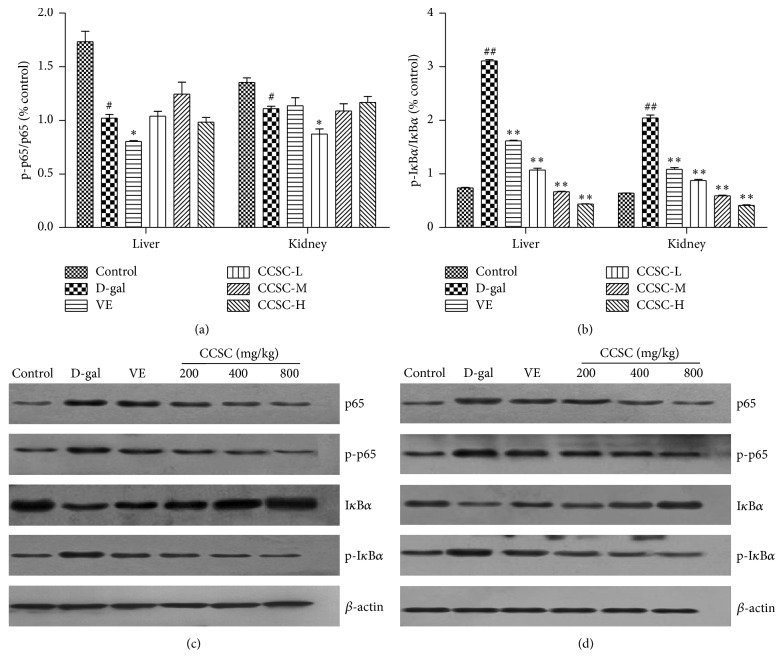
Effect of CCSC on the protein expression of NF-*κ*B pathway. Relative density analysis of p-p65/p65 (a) and p-I*κ*B*α*/I*κ*B*α* (b) and the representative images of p-I*κ*B*α*, I*κ*B*α*, p-p65, and p65 expression in the liver (c) and kidney (d) tissues. The relative protein level between the tested target protein and internal standard *β*-actin was calculated and labeled on the *y*-axis. The bands are from a representative blot. Lane-1: control group; lane-2: D-gal-treated group; lane-3: VE-treated group (80 mg/kg); lane-4: CCSC-L-treated group (200 mg/kg); lane-5: CCSC-M-treated group (400 mg/kg); lane-6: CCSC-H-treated group (800 mg/kg). Values are expressed as mean ± SD in each group. ^#^ *P* < 0.05 and ^##^ *P* < 0.01 as compared with the control group; ^*∗*^ *P* < 0.05 and ^*∗∗*^ *P* < 0.01 as compared with the D-gal group, *n* = 3.

The expression of iNOS, COX2, I*κ*B*α*, p-I*κ*B*α*, p65, and p-p65 in total proteins of the liver and kidney tissues of D-gal-treated mice was analyzed by western blotting analysis. As shown in Figure 4, the protein levels and the mean optical densities of iNOS and COX2 were higher in the D-gal-treated mice than the control group. However, CCSC and VE treatments caused a reduction in the protein levels and the mean densities of iNOS and COX2 in a dose-dependent manner. In Figure 5, our results clearly exhibited that CCSC group significantly increased I*κ*B*α* level and decreased levels of p-I*κ*B*α*, p65, and p-p65 in D-gal-treated liver and kidney tissues with the decreased ratio of p-I*κ*B*α* to I*κ*B*α*. However, p-p65 versus p65 exhibited no significant differences both in liver and kidney tissues compared with D-gal group. Furthermore, our result showed that CCSC group in liver and kidney all showed more potent effect in downregulating the protein expression of iNOS, COX2, p65, p-p65, and p-I*κ*B*α* and upregulating I*κ*B*α* expression than VE (Figures 4(c), 4(d), 5(c), and 5(d)). It was noteworthy that high dose of CCSC-H (800 mg/kg) group exhibited particularly strong effect in downregulating and upregulating the protein expression of iNOS, COX2, p65, p-p65, p-I*κ*B*α*, or I*κ*B*α*, restoring these protein levels almost to the level of the control group in both liver and kidney.

### 3.7. Histopathological Analysis

**Figure 6:**
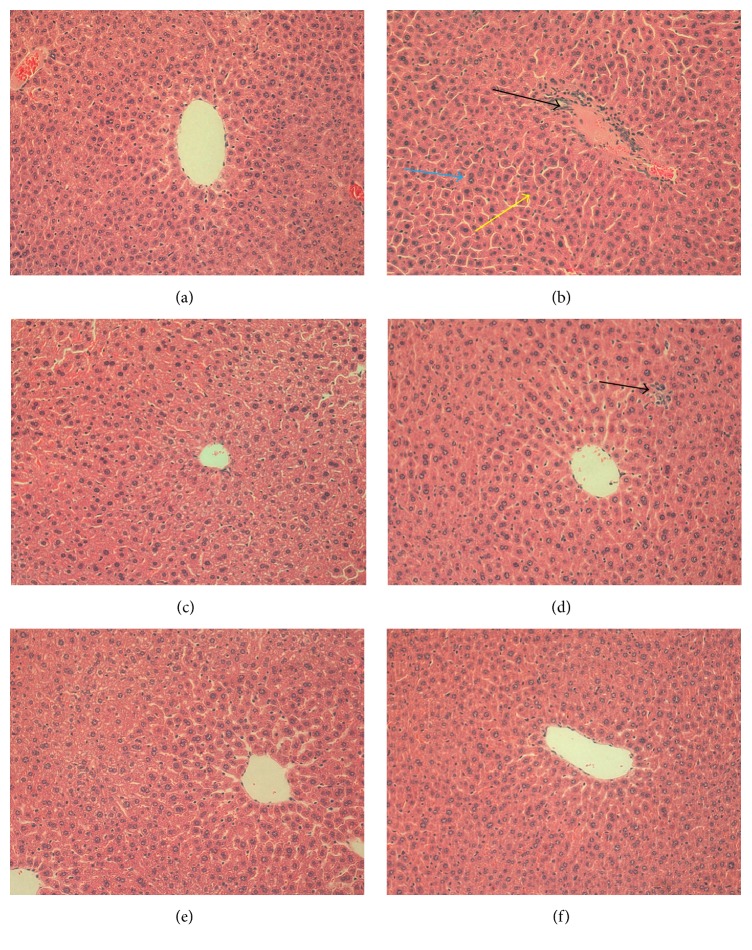
Histopathological appearance of liver in groups (H&E staining 200x). (a) Control group, (b) D-gal (200 mg/kg) group, (c) VE (80 mg/kg) group, (d) CCSC-L (200 mg/kg) group, (e) CCSC-M (400 mg/kg) group, and (f) CCSC-H (800 mg/kg) group. Inflammatory infiltration (black arrow), hepatic cord arranged mussily (yellow arrow), and binucleation of hepatocytes (blue arrow).

**Figure 7:**
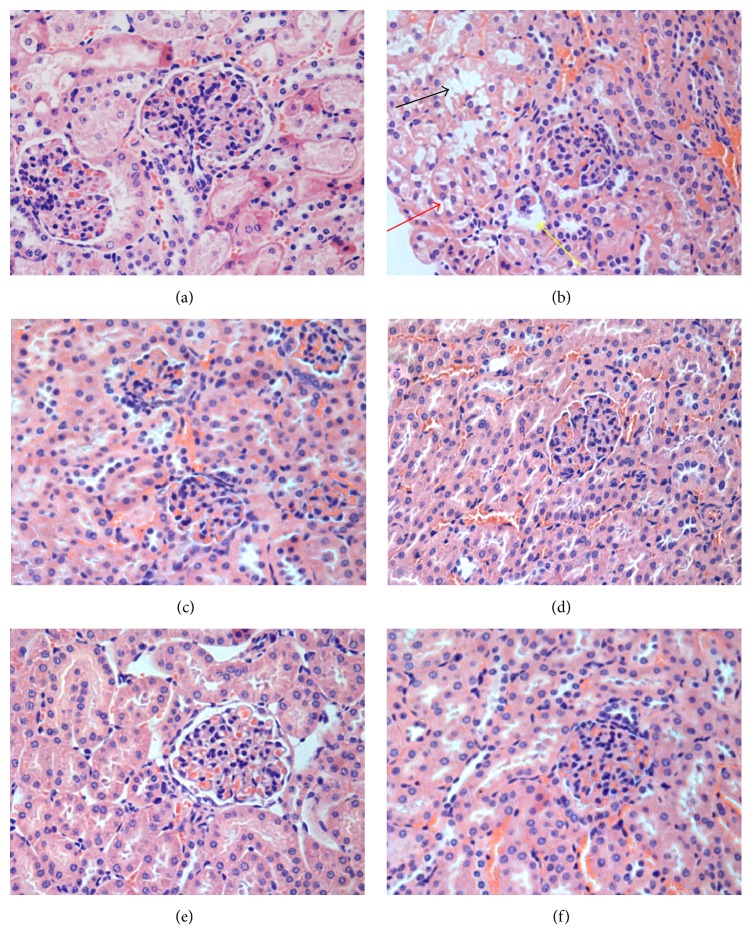
Histopathological appearance of kidney (H&E staining 400x). (a) Control group, (b) D-gal (200 mg/kg) group, (c) VE (80 mg/kg) group, (d) CCSC-L (200 mg/kg) group, (e) CCSC-M (400 mg/kg) group, and (f) CCSC-H (800 mg/kg) group. Atrophy (yellow arrow), balloon widened cavity (red arrow), and drop of epithelial cells in renal proximal convoluted tubules (black arrow).

In the D-gal-treated mice, the histopathological examination of liver tissue revealed mussily arranged hepatic cord, binucleation of hepatocytes, and a large number of inflammatory cell infiltrations when compared to the control group (Figure 6(a) **)**. However, in CCSC treatment groups, neatly arranged hepatic cord, fewer inflammatory infiltrations, and binucleation of hepatocytes were observed in liver tissues (Figures 6(d), 6(e), and 6(f)). On the other hand, the histopathological examination of kidney tissue showed that, in D-gal group, glomerulus showed obviously atrophy or even disappeared, balloon widened cavity, and drop of epithelial cells could occur in renal proximal convoluted tubules as compared with control group. However, CCSC and VE treatment significantly attenuated the glomerular atrophy and decreased the balloon widened cavity (Figures 7(c), 7(d), 7(e), and 7(f)).

## 4. Discussion

It has been shown that D-gal-treated mice were found similar to those of natural aging [23]. At high levels, D-gal could cause the metabolism of sugar in disorder and lead to the accumulation in the cell and induce osmotic stress and produce reactive oxygen species (ROS). Oxidative damage caused by reactive oxygen species (ROS) is expected to play a role in age-related changes in tissues, such as liver, brain, and kidney. D-gal treatment was also reported to cause inflammatory reactions and apoptotic and necrotic changes in the organs of animals and therefore has been widely used to establish an aging model for antiaging research and organ injured model [5, 24, 25].

It was reported that different ingredients potentiated each other's effect [26]. In traditional Chinese medicine, plant extracts from different herbs in a formula may contain different ingredients, which may play a different role in treating the same disease. CCSC, a traditional Chinese medicine compound recipe consisting of five food material and medicine herbs, including Misai Kuching, North Cordyceps, Longan Arillus, Folium Ginseng, and Corni Fructus, has various pharmacological effects. Misai Kuching, a well-known medicine, is commonly used as herbal tea for diuresis, to treat rheumatism, diabetes, urinary lithiasis, oedema, eruptive fever, influenza, hepatitis, jaundice, biliary lithiasis, and hypertension [27, 28]. North Cordyceps, a precious medicinal herb widely distributed in China, is high in medicinal and nutritious components than Cordyceps and easy in cultivation and cheaper than Cordyceps [29]. North Cordyceps is widely used in functional food and healthcare products in China and exhibits various bioactive effects including regulating the immune function, invigorating the kidney, inhibition of cancer cell, deferring consenescence, and increasing storage of glycogen and antifatigue [30]. HPLC-ESI-MS/MS results indicated that CCSC consisted of many components, such as uridine, cordycepin, loganin, rosmarinic acid, ginsenoside Rg1, and Re. Zhang et al. found that rosmarinic acid has effect on liver and kidney antioxidant enzymes, lipid peroxidation, and tissue ultrastructure in aging mice [20]. Cordycepin attenuated age-related oxidative stress and ameliorated antioxidant capacity in rats [31]. Loganin exhibited anti-inflammatory effect via inhibiting NF-*κ*B activation [21]. Uridine and ginsenosides Rg1 and Re were also found to have various effects, such as antioxidant and antifatigue [19, 22, 32, 33].

Kidney and liver are two important organs in metabolism system. Their functions are declined gradually due to their structure atrophy with age. Recent studies showed that chronic administration of D-gal induced a mimetic aging effect in various tissues of rodents, such as liver, kidney, and brain. For the immune system, thymus and spleen, two important immune organs, also presented physiological diminution by D-gal treatment [34]. The present study clearly demonstrated that subcutaneous injection of D-gal at dose of 200 mg/kg/day for 8 weeks caused a severe aging-related appearance changes, including significant decrease in body weights and organ indexes. Meanwhile, kidney and liver were atrophied in D-gal-treated mice. However, CCSC supplement could partially reverse these deteriorating effects, increase both body weights and organ indexes, and as improve the histopathologies of liver and kidney. The abovementioned observation suggested that CCSC could alleviate liver and kidney injury in D-gal-treated mice and the underlying molecular mechanisms were explored and described as follows.

Previous studies have shown that treatment with D-gal causes liver injury and dysfunction, followed by elevated activities or levels of serum enzymes [35]. AST, ALT, BUN, and CRE are commonly deemed as the key injury indicators for the liver and kidney [36–38]. The present study showed that AST, ALT, BUN, and CRE had higher levels in aged mice compared to the control group, which was in line with the previous report [39]. However, these increased levels were significantly decreased by CCSC and VE treatment, suggesting the protective effect of CCSC against D-gal-induced liver injury.

Oxidative stress could increase the generation of free radicals and could impair the antioxidant enzymes. A large number of studies revealed that the balance between ROS system and autoxidation system determined the degree of oxidative stress [40]. SOD is a superoxide radical scavenging factor that converted superoxide radicals to H_2_O_2_ [41]. CAT catalyses the decomposition of H_2_O_2_ into H_2_O and O_2_ [42] and GPx reduces H_2_O_2_ or hydroperoxides to H_2_O and alcohol [42] where they are regarded as the first line of defense against the ROS generated during oxidative stress [43]. Meanwhile, MDA is one product of lipid peroxidation, whose content reflects the damage to the cell membrane [41]. The liver is a major organ involved in D-gal metabolism and D-gal treatment is found to increase hepatic MDA levels and cause DNA damage together with oxidative stress [5]. In the present work, subcutaneous injection of D-gal caused the oxidative stress, decreased the antioxidant enzymatic activity of SOD, CAT, and GPx, and increased the MDA level. However, supplementation of CCSC was observed to decrease the MDA level and restore the antioxidant defense system by increasing the activity of antioxidant enzymes and modulating the hepatic mRNA expressions of antioxidant enzymes in the livers of D-gal-treated mice in a dose-dependent manner, even stronger than the typical antioxidant VE. Taken together, CCSC administration protected the mouse liver against D-gal-induced hepatocyte oxidative stress via elevating multiple antioxidants gene expression and enhancing the antioxidant capacity. The results indicated that CCSC with antioxidant property might act as a potential candidate applicable for the antioxidant based functional formulae in complimentary or integrated therapy of age-related diseases.

On the one hand, oxidative stress could induce direct cellular and tissue damages; on the other hand, oxidative stress could activate transcription factors including NF-*κ*B pathway which regulate the expression of various inflammatory genes that could determine and maintain low-grade inflammation during aging and age-associated diseases [13]. The p65 protein is a key active component of NF-*κ*B. In most types of cells, NF-*κ*B dimers are transcriptionally inactive in cytoplasm due to the inhibitors of three I*κ*B isoforms (I*κ*B*α*, I*κ*B*β*, and I*κ*B*ε*) [44]. I*κ*B degradation allows NF-*κ*B to translocate to the nucleus and bind DNA. In addition, under certain circumstances, NF-*κ*B dimers are activated and promote the phosphorylation of I*κ*B. The activated NF-*κ*B is translocated to the nucleus and regulates the proinflammatory gene expression such as iNOS and COX2 related to inflammatory, immune, fibrogenic, carcinogenic, and acute phase responses so as to cause tissue and organ damage following severe trauma [45, 46]. COX2-mediated inflammatory responses play important roles in biology and diseases such as renal function, nerve and brain function, ovarian and uterine function [47]. iNOS can consistently release high levels of nitric oxide (NO) and results in deleterious effects in both local and systemic inflammatory responses [48]. Therefore, inhibition of COX2, iNOS, and NF-*κ*B expression may be an effective strategy to suppress the inflammatory responses. The present study showed that CCSC treatment not only significantly downregulated the protein expression of p-I*κ*B*α*, NF-*κ*B p65, p-p65, iNOS, and COX2, but also obviously upregulated the I*κ*B*α* level in mice liver and kidney (Figures 4 and 5), suggesting that CCSC might exert anti-inflammatory effect via blocking the activation of NF-*κ*B signaling pathway and inhibiting iNOS and COX2 expression.

## 5. Conclusion

The present study demonstrated that CCSC could attenuate D-gal-induced liver and kidney injury in mice and the mechanism might be intimately associated with attenuating lipid peroxidation, renewing the activities of antioxidant enzymes, enhancing the antioxidant enzyme gene expression, and suppressing inflammatory response.

## Figures and Tables

**Table 1:** Primer sequences.

Gene	Sequence	Product size	Accession number
18S RNA	CCTGGATACCGCAGCTAGGA	112 bp	NR_003286
	GCGGCGCAATACGAATGCCCC		

SOD1	GTCGGCTTCTCGTCTTGCTC	80 bp	NM_011434.1
	GCTTTCATCGCCATGCTTCC		

CAT	CGTCCGTCCCTGCTGTCTCA	109 bp	NM_009804.2
	CTGCTCCTTCCACTGCTTCATC		

GPx1	CGGGACCCTGAGACTTAGAGC	191 bp	NM_008160.6
	GAAGGCATACACGGTGGACTG		

The constitutive gene 18S was used as an internal control.

**Table 2:** Peak assignment of aqueous extracts of CCSC using HPLC-ESI-MS/MS in positive and negative ionization modes.

Number	RT (min)	Molecular weight	HPLC-ESI-MS		Fragment ions	Identified compounds
			[M + H]^+^ * m/z*	[M − H]^−^ * m/z*		
1	7.8	198.05	—	197.04	135, 123	Salvianic acid
2	8.6	170.02	—	169.01	125, 107	Gallic acid
3	9.5	244.07	—	243.06	200, 152, 147, 110	Uridine
4	12.0	283.09	—	282.08	150, 133	Guanosine
5	21.6	251.10	252.11	—	136, 119	Cordycepin
6	34.3	388.14	389.14	—	227.1, 209, 195.1, 149	Verbenalin
7	37.0	180.04	—	179.03	135, 117	Caffeic acid
8	38.6	270.05	—	269.04	251, 179, 161, 135	Baicalein
9	42.6	390.15	—	389.14	227, 127	Loganin
10	44.9	358.13	359.13	—	197, 179, 151, 127	Sweroside
11	48.3	360.08	—	359.07	197, 179, 161, 135, 123	Rosmarinic acid
12	83.9	472.36	473.36	—	464, 416, 360, 351	2*α*-Hydroxy-ursolic acid
13	84.9	800.49	—	799.48	637, 475.4	Ginsenoside Rg1
14	84.9	946.55	—	945.54	783, 637, 475	Ginsenoside Re
15	97.7	374.10	—	373.09	179, 135	Methyl rosmarinate

**Table 3:** Effects of CCSC on the body weight and organ index in D-gal-treated mice (mean values and standard deviations, *n* = 10 per group).

Group	Weight (g)		Organ index (mg/g)			
	Initial	Final	Liver	Kidney	Thymus	Spleen
Control	20.9 ± 1.29	34.21 ± 1.51	3.64 ± 0.21	1.04 ± 0.09	1.95 ± 0.23	3.33 ± 0.13
D-gal	20.0 ± 0.68	30.58 ± 1.09^##^	3.29 ± 0.08^##^	0.93 ± 0.09^##^	1.49 ± 0.19^##^	2.80 ± 0.10^##^
VE	20.1 ± 0.85	32.98 ± 1.49^*∗∗*^	3.60 ± 0.25	1.03 ± 0.11^*∗*^	1.92 ± 0.35^*∗∗*^	3.11 ± 0.21^*∗∗*^
CCSC-L	19.7 ± 0.62	33.28 ± 1.36^*∗∗*^	3.65 ± 0.25^*∗*^	1.02 ± 0.08^*∗*^	1.70 ± 0.34	3.04 ± 0.12^*∗∗*^
CCSC-M	20.1 ± 0.35	33.76 ± 1.71^*∗∗*^	3.68 ± 0.34	1.05 ± 0.07^*∗∗*^	1.99 ± 0.22^*∗∗*^	3.05 ± 0.11^*∗∗*^
CCSC-H	19.9 ± 0.67	33.69 ± 1.61^*∗∗*^	3.55 ± 0.25	1.07 ± 0.09^*∗∗*^	1.78 ± 0.23^*∗*^	3.26 ± 0.26^*∗∗*^

^##^ *P* < 0.01 compared to the control group. ^*∗*^ *P* < 0.05 and ^*∗∗*^ *P* < 0.01 compared to the D-gal group.

**Table 4:** Effects of CCSC on liver and kidney functions in D-gal-treated mice.

	Liver function		Kidney function	
	ALT (U/L)	AST (U/L)	BUN (*μ*moL/L)	CRE (*μ*moL/L)
Control	31.48 ± 0.85	107.54 ± 2.75	5.44 ± 0.39	25.63 ± 2.03
D-gal	64.41 ± 1.48^##^	163.62 ± 2.04^##^	16.47 ± 0.49^##^	82.36 ± 2.45^##^
VE	37.21 ± 1.00^*∗∗*^	111.44 ± 3.13^*∗∗*^	9.78 ± 0.62^*∗∗*^	43.00 ± 2.59^*∗∗*^
CCSC-L	36.93 ± 0.99^*∗∗*^	112.10 ± 3.06^*∗∗*^	11.01 ± 1.24^*∗∗*^	43.88 ± 3.19^*∗∗*^
CCSC-M	40.72 ± 1.09^*∗∗*^	117.14 ± 2.74^*∗∗*^	9.38 ± 0.51^*∗∗*^	41.74 ± 1.99^*∗∗*^
CCSC-H	43.73 ± 1.17^*∗∗*^	119.55 ± 2.89^*∗∗*^	8.89 ± 0.85^*∗∗*^	35.15 ± 2.81^*∗∗*^

Mean values and standard deviations, *n* = 10 per group. ^##^ *P* < 0.01 compared to the control group. ^*∗∗*^ *P* < 0.01 compared to the D-gal group.
